# Facing SpO_2_ and SaO_2_ discrepancies in ICU patients: is the perfusion index helpful?

**DOI:** 10.1007/s10877-019-00371-3

**Published:** 2019-08-07

**Authors:** Mark Thijssen, Loes Janssen, Jos le Noble, Norbert Foudraine

**Affiliations:** 1 Department of Intensive Care, VieCuri Medical Center, Tegelseweg 210, 5912BL Venlo, The Netherlands; 2 Department of Statistics and Epidemiology, VieCuri Medical Center, Tegelseweg 210, 5912BL Venlo, The Netherlands

**Keywords:** Critical illness, Intensive care unit, Oxygen saturation, Perfusion index, Pulse oximetry

## Abstract

Peripheral oxygen saturation (SpO_2_) measured by pulse oximetry is an unreliable surrogate marker for arterial oxygenation (SaO_2_) in critically ill patients. We hypothesized that a higher perfusion index (PFI) would be associated with better accuracy of SpO_2_ measurement. We retrospectively collected SaO_2_, SpO_2_, and PFI data for each arterial blood gas (ABG) analysis in a cohort of intensive care unit patients. PFI was categorised as low (PFI < 1.0), intermediate (1.0 ≤ PFI  ≤ 2.5), or high (PFI > 2.5). The correlation between SpO_2_ and SaO_2_ was studied using Pearson’s correlation. The Bland–Altman plot was used to analyse the agreement between SpO_2_ and SaO_2_. Furthermore, the correlation between the (SpO_2_–SaO_2_) difference and PFI was assessed. The level of (dis)agreement was calculated for the three PFI categories separately. Overall, 281 patients and 1281 data points were analysed. There was a significant correlation between SaO_2_ and SpO_2_ (r = 0.69, p < 0.01). The Bland–Altman analysis revealed a mean difference between SaO_2_ and SpO_2_ of 0.2% with limits of agreement of ± 6% (SD ± 2%). The correlation between the PFI and the (SpO_2_–SaO_2_) difference was low; the (SpO_2_–SaO_2_) difference improved only marginally with higher PFI values. The accuracy of pulse oximetry for estimating arterial oxygenation was moderate and improved little with increasing PFI values. Thus, the additive value of PFI in clinical decision making is limited. Therefore, we advise performing an ABG before adjusting fraction of inspired oxygen (FiO_2_) settings.

## Introduction

Pulse oximetry is routinely used to monitor peripheral oxygen saturation (SpO_2_) as a surrogate marker for arterial oxygen saturation (SaO_2_). Strict guidelines regarding an optimum target SpO_2_ are lacking. However, most clinicians will aim for SpO_2_ values between 88 and 96%, depending on the clinical situation, and might surmise that the real arterial oxygenation is above the measured values of the pulse oximeter [1]. However, one can erroneously accept lower arterial oxygenations than surmised. Although it is a useful method for monitoring intensive care unit (ICU) patients, it has limitations. In the critically ill, complex physiological disturbances such as altered blood flow, acid–base disturbances, and abnormalities in temperature regulation may occur; these affect the oxyhaemoglobin dissociation curve and might further complicate the interpretation of the SpO_2_ [2]. Moreover, the accuracy of a pulse oximeter is reduced in patients with low perfusion status, sepsis, and vasopressor use [3, 4]. Consequently, the new oximeters can calculate the perfusion index (PFI) from a pulsatile photo plethysmography signal and indirectly measure the perfusion variations. The PFI may function as a marker for peripheral perfusion and resembles vasomotor tone, with low and high PFI values indicating perfusion below and above average, respectively [5–9]. In routine ICU practice, the PFI could possibly be used as a surrogate marker for the accuracy of measured SpO_2_ [10].

The primary objective of our study was to assess whether, in a cohort of ICU patients, the use of the PFI in daily practice contributes to the accuracy of pulse oximetry. We hypothesized that higher PFI values would be associated with more accurate SpO_2_ measurement. Therefore, we studied the relationship between SaO_2_ and SpO_2_, and how this depended on the PFI. Furthermore, as a secondary objective, we tested whether multiple variables (temperature, MAP, pH, lactate, and inotropic drug use) influenced the (SpO_2_–SaO_2_) difference and PFI.

## Materials and methods

We performed a retrospective study in a level 2 mixed ICU of a single-site teaching hospital. Patients were included in the study between May 2015 and September 2015. All admitted ICU patients on supplemental oxygen therapy, with at least one arterial blood gas (ABG) analysis and concomitantly measured SpO_2_, were eligible for inclusion.

### Data collection

Data regarding patient characteristics, admission diagnosis, length of stay (LOS), days on non-invasive/invasive mechanical ventilation, SpO_2_, PFI, and variables that could potentially influence pulse oximetry measurements such as central body temperature, mean arterial pressure (MAP), pH, lactate, and inotropic drug use were retrieved from the electronic patient data management system (EPD). EPD data verification was performed by the research nurse. In patients treated with invasive ventilation, all ABG samples were included until extubation. In all other cases, one daily ABG was included until a maximum of 3 days after ICU admittance in order to limit the total amount of data, under the assumption that any further data would not strongly influence outcomes.

SaO_2_, PaO_2_, pH, and lactate were measured with a blood gas analyser (ABL 800 Flex, Radiometer, Copenhagen, Denmark) in the local clinical chemistry department; this method is considered the gold standard.

### Pulse oximeters

SpO_2_ was measured continuously; however, for this study, SpO_2_ registered only at the time of blood withdrawal using the Philips M1191BL finger probe (Philips Healthcare, Eindhoven, The Netherlands) and the Philips M1194A ear probe (Philips Healthcare, Eindhoven, The Netherlands). According to the manufacturer, these pulse oximeters have an accuracy of ± 2.5% and ± 4% root mean square (RMS), respectively [11]. Both pulse oximeters were connected to the Philips MP70 monitor (Philips Healthcare, Eindhoven, The Netherlands) [10].

### Statistical analysis

All statistical analyses were performed with IBM SPSS Statistics version 23 (IBM Corp. Released 2015. IBM SPSS Statistics for Windows, Version 23.0. Armonk, NY: IBM Corp.) Continuous data are presented as a mean if normally distributed and as a median otherwise. Categorical data are presented as percentages.

#### Accuracy of pulse oximetry

We used the Pearson correlation coefficient (r) to express the relationship between SaO_2_ and SpO_2_. The Bland–Altman plot was used to graphically represent this relationship [12]. With this method, the difference between SpO_2_ and SaO_2_ (ΔSat) was plotted against the average of SpO_2_ and SaO_2_. A positive value indicated that the measured SaO_2_ was lower than SpO_2_. Furthermore, regression analysis was performed to determine whether there was positive, negative, or no correlation between the bias and the average [13]. The Pearson correlation coefficient was used to express the possible association of temperature, pH, lactate, and MAP with ΔSat. Furthermore, a Student’s *t* test was performed to test the association of sex with ΔSat. One-way ANOVA was used to test the association of inotropes with ΔSat.

#### The perfusion index

To evaluate the relationship between the (skewed) PFI and ΔSat, the Spearman correlation coefficient was calculated. Subsequently, we determined whether the PFI is a reliable surrogate for the accuracy of the pulse oximeter. We categorised PFI values as low (PFI < 1.0), intermediate (1.0 ≤ PFI ≤ 2.5), or high (PFI > 2.5) [5, 7, 10]. Accuracy was visualised by plotting ΔSat against the PFI. In addition, we calculated the percentages of measurements exceeding various limits of agreement for the three PFI categories.

The study protocol was approved by the local Ethics Review Board and only the primary investigator was able to link the study data with the patient data [14].

## Results

A total of 320 patients were eligible for the study. The data from 39 patients were missing and the remaining 281 were included in the analysis, resulting in 1281 data points. Baseline patient characteristics are shown in Table 1.

**Table 1:** Standard patient characteristics

Patient characteristics (n = 281)	n (%)
Sex	
Male	177 (63)
Female	104 (37)
Age in years (SD)	65 (15)
Diagnosis at admittance*	
Pneumonia	31 (11.0)
Cardiac arrest	28 (10.0)
Trauma	23 (8.2)
GI bleeding	18 (6.4)
Sepsis	16 (5.7)
COPD exacerbation	15 (5.3)
Length of stay in days (range)	2 (0–67)
Days intubated (range)	3 (0–58)
Inotropic use	
Norepinephrine	102 (36)
Dobutamine	10 (4)
Combination	19 (7)
APACHE IV*	87 (35)
SaO_2_ in % (SD)*	96.1 (3.8)
SpO_2_ in % (SD)*	96.3 (3.9)
PFI in % (range)*	1.4 (0.1–19.2)

Continuous data are presented as means (SD) or medians (interquartile range). Categorical data are presented as percentages

*GI Bleeding gastrointestinal bleeding, *COPD* chronic obstructive pulmonary disease, *APACHE IV* acute health and chronic health evaluation IV, *SaO*_*2*_ arterial oxygen saturation, *SpO*_*2*_ peripheral oxygen saturation, *PFI* perfusion index

**Fig. 1:**
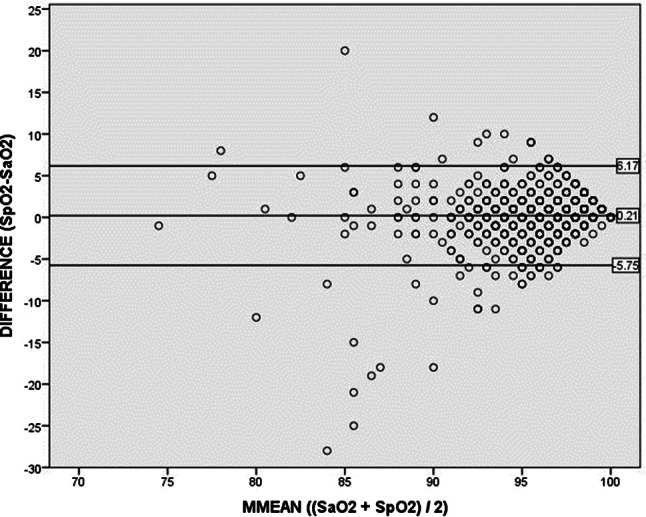
Bland–Altman plot, in which the difference between SpO_2_ and SaO_2_ is plotted against their average. The mean difference is 0.21% (middle line), with a SD (precision) of 3.04%. Limits of agreement were calculated using mean ± (1.96 * SD), resulting in an upper limit of 6.17% and a lower limit of − 5.75% (outer lines)

On average, SaO_2_ and SpO_2_ correlated moderately (r = 0.69, p < 0.01, n = 1281). The Bland–Altman plot revealed a mean ΔSat of 0.21%, with a standard deviation (SD) of 3.04% (Fig. 1). Limits of agreement within 2 SDs of the mean showed a ΔSat of − 5.75% and + 6.17%. Furthermore, a non-significant linear regression between the bias (ΔSat) and the magnitude of the measurements (mean of SaO_2_ and SpO_2_) was found (F (1,1279) = 1.44, p < 0.23), with a R^2^ of 0.001, indicating no correlation between the bias and the error and verifying reliable use of the Bland–Altman analysis.

The mean ΔSat was not different between males and females (0.27 versus 0.13, p = 0.42) and was not associated with inotropic drugs use (F (3,1277) = 0.94, p = 0.42). In addition, other independent variables showed no or poor association with ΔSat (pH: r = − 0.18, p < 0.01; lactate: r = 0.02, p = 0.60; MAP: r = − 0.006, p = 0.82).

In the case of the largest measured ΔSat, we measured both methaemoglobin (MetHb) and carboxyhaemoglobin (COHb). All values of combined MetHb plus COHb were under 2% (data not shown).

### The perfusion index

**Fig. 2:**
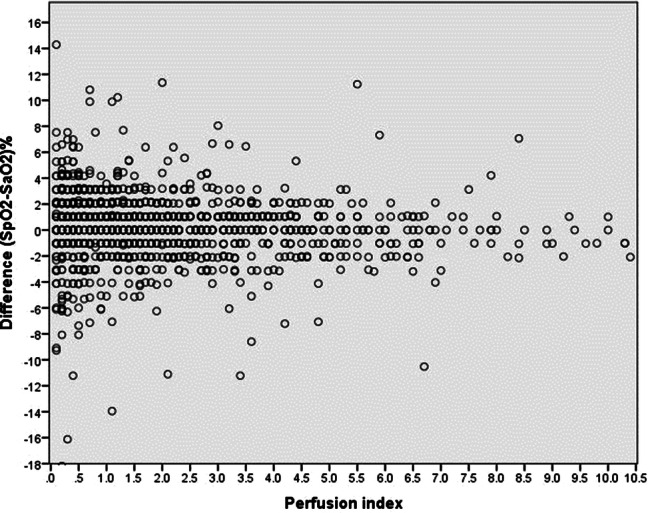
The ΔSat as percentages ((SpO_2_–SaO_2_/SaO_2_) × 100%) plotted against the PFI

PFI and ΔSat correlated weakly (r = − 0.17, p < 0.01), indicating a slightly better agreement between SaO_2_ and SpO_2_ with increasing PFI (Fig. 2, Table 2). A large proportion of values exceeded the limits of agreement, which did not decrease drastically with increasing PFI. For example, even with a PFI > 2.5, which is generally considered adequate, 15.9% of all measurements still showed a difference between SpO_2_ and SaO_2_ of more than 2% (Table 2). Thus, the effect of PFI on ΔSat is limited.

**Table 2:** Percentages and datapoints (n) of measurements exceeding varying limits of agreements (columns) for the various PFI categories (rows)

ΔSat (SpO_2_–SaO_2_) PFI	> 1%	> 2%	> 3%	> 4%
< 1.0	52.8% (246)	30.7% (143)	18.7% (87)	10.5% (49)
1.0–2.5	38.3% (153)	18.3% (73)	8.5% (34)	4.8% (19)
> 2.5	36.2% (130)	15.9% (57)	6.9% (25)	4.1% (15)

## Discussion

We performed a retrospective, single-centre study to demonstrate the contribution of the PFI to the accuracy of pulse oximetry in critically ill adult patients. The main finding of our study is that even with high PFI values, the accuracy of pulse oximetry in estimating arterial oxygenation remains moderate; therefore, the additive value of PFI is limited. In our study, pulse oximetry and SaO_2_ measurements correlated moderately (r = 0.69). Even when the outliers were removed, results remained identical. As the correlation coefficient found between SaO_2_ and SpO_2_ was 0.69, only 50% of the variability of SpO_2_ could be explained by the difference in SaO_2_. This is partly explained by a 3% variability of the pulse oximeter, compared to a variability of 0.2% of the blood gas analyser. This indicates that various other factors, such as acid–base disorders or factors contributing to macro- or microcirculation, might play a role in determining the absolute values of both SpO_2_ and SaO_2_. This comparison between SpO_2_ and SaO_2_ was also substantiated in the Bland–Altman plot, showing only moderate accuracy with varying clinical conditions.

Our findings regarding a discrepancy between SaO_2_ and SpO_2_ measurements are in line with the results of previous studies conducted with similar cohorts of critically ill patients. Perkins et al. demonstrated that changes in SpO_2_ do not reliably predict changes in SaO_2_, and neither anaemia nor acidosis altered the relationship between SpO_2_ and SaO_2_ [15]. We only found a small and weak negative correlation between the pH and ΔSat. In a cohort of ventilator-dependent patients, Seguin et al. showed that SpO_2_ overestimated SaO_2_, and a minimum SpO_2_ value of 96% to ensure SaO_2_ > 90% was incorporated into a nurse-driven protocol [16]. In a study by van de Louw et al., large differences between SpO_2_ and SaO_2_ were found with poor SpO_2_ reproducibly. Both studies suggested an SpO_2_ above 94% to ensure SaO_2_ > 90% [16, 17]. The accuracy of pulse oximetry was studied by Wilson et al. in a cohort of septic patients admitted to the emergency department. Their main finding was that pulse oximetry overestimated ABG-determined SaO_2_ by 2.75% [4]. When SaO_2_ needed to be determined, ABG analysis was recommended. A key problem in interpreting the findings reported in the literature on the use of pulse oximetry is that different kinds of pulse oximeters are used with different patented techniques for calculating SpO_2_, making comparisons difficult [18]. Corrections for the PFI have not been made in any of these studies.

Several possible explanations can be put forward to explain the disparity between the ΔSat and the PFI values. Vasodilation in skin and muscle is not equally distributed in acid–base disorders. Higher pH results in vasodilation in the muscle but vasoconstriction in skin arteries where the SpO_2_ measurements are taken [19]. This skin vasoconstriction may result in lower SpO_2_ values and therefore the ΔSat (SpO_2_–SaO_2_) may decrease, resulting in a negative correlation. Moreover, a lower pH results in a lower SaO_2_ due to the rightward shift of the oxygen dissociation curve (Bohr Effect), resulting in an increased ΔSat. Furthermore, in general and in isolated arteries, a lower pH results in vasodilation and therefore higher PFI values [19]. This association might result in a negative correlation. However, in our patient group a positive correlation was found. This might be explained by the fact that acidotic patients are frequently hemodynamically less stable, have a lower cardiac output, and are at risk for higher inotropic use. These factors might independently contribute to a lower PFI. In conclusion, many interacting factors may result in vasodilation or vasoconstriction. Our results have small correlation coefficients and may result in divergent values of blood oxygen levels.

To our knowledge, this is the first clinical study that correlated SpO_2_ accuracy with the PFI in adults. However, there are several limitations to our study. In our ICU, the ABG measurements were performed in our local central laboratory rather than with point-of-care equipment. A delay in the laboratory measurements could result in a lower arterial oxygen content and thus increased frequency of a positive SpO_2_–SaO_2_ difference. In our analyses this was not found, so we believe that this may not have influenced our results significantly. A second point of concern might be that we only used Philips pulse oximetry equipment, making head-to-head comparison with other manufacturers and extrapolation to different clinical settings difficult. However, a study by Louie et al. demonstrated that the Philips, Masimo, and Nellcor pulse oximeters were similarly effective in detecting hypoxemia [18]. Furthermore, besides the surmised but not proven value concerning the reliability of the linear relationship between SpO_2_ and SaO_2_, PFI values have also been used to predict vasopressor requirements or mortality [2, 20–23]. Clearly, differences in patient population may account for this, and our study was neither powered nor designed to assess inotropic drug use or mortality. Another possible drawback of our study is that it was conducted in a single centre; however, bias can also be introduced easily in a multi-centre trial as equipment and laboratory procedures may differ slightly.

Given that SpO_2_ does not reliably predict SaO_2_ values, despite in accordance to the manufacturer’s precision (both around 2–3%), the question arises as to how supplemental oxygen therapy can be adjusted without creating a hypoxic or hyperoxic state [11]. For example, in our ICU, we would rather be informed if the patient has an SaO_2_ of 89% instead of 93%, whereas the corresponding monitor indicates 91%. Moreover, our results imply that the PFI is not an accurate marker for SaO_2_ extrapolation and therefore will not be the primary determinant for adjusting the fraction of inspired oxygen (FiO_2_) values to improve supplemental oxygen delivery. Hence, we suggest performing an ABG analysis to measure SaO_2_.

## Conclusions

Our results indicate a clinically relevant discrepancy between SaO_2_ and SpO_2_ and only a small decrease in their difference in the presence of high arterial perfusion. However, even at high PFI values, differences in saturation can still be clinically significant. These findings may influence daily practice on how to adjust oxygen supply therapy based on SpO_2_ measurements only. In critical situations, we advise collecting ABG measurements instead of adjusting oxygen supply based on SpO_2_ values, regardless of the PFI values.
